# Impact of Connected Mental Health on the Work Environment of Mental Health Clinicians: Protocol for a Systematic Literature Review

**DOI:** 10.2196/76668

**Published:** 2025-10-27

**Authors:** Shweta Premanandan, Sofia Ouhbi, Magdalena Ramstedt Stadin, Charlotte Blease, Åsa Cajander, Maria Hägglund

**Affiliations:** 1 Division of Computing Science Department of Information Technology Uppsala University Uppsala Sweden; 2 Vi3 Department of Information Technology Uppsala University Uppsala Sweden; 3 Participatory eHealth and Health Data Research Group Department of Women's and Children's Health Uppsala University Uppsala Sweden

**Keywords:** connected mental health, work environment, mental health clinicians, digital mental health applications, burnout, systematic literature review, artificial intelligence

## Abstract

**Background:**

Many mental health professionals face work-related stress due to high job demands, limited control, and inadequate institutional support. Connected mental health (CMH) technologies such as mobile apps and teletherapy platforms are increasingly being proposed as tools to alleviate these job demands. However, their actual influence on clinicians’ work environments—here understood as the organizational, social, and psychological conditions that shape their workload, job demands, autonomy, and overall well-being—remains underexplored. Existing reviews have primarily focused on traditional organizational interventions, leaving a critical gap in understanding how CMH technologies specifically influence the work environment of mental health clinicians.

**Objective:**

This systematic literature review aims to identify and summarize knowledge about the impact of CMH on the work environment of mental health clinicians.

**Methods:**

A systematic literature review will be performed. The review follows PRISMA (Preferred Reporting Items for Systematic Reviews and Meta-Analyses) guidelines and has been registered in PROSPERO on April 23, 2025. A comprehensive search strategy was developed using the population, intervention, comparison, and outcome (PICO) framework in collaboration with an academic librarian. Studies will be sourced from the PubMed, Scopus, IEEE Xplore, and ACM Digital Library databases. Inclusion criteria are limited to empirical studies involving mental health clinicians using CMH tools, where outcomes explicitly relate to the work environment (eg, job demands, workload, autonomy, stress, or well-being). Eligible studies must be published in English. Data extraction will include publication trends, study methods, and types of CMH technologies. Additionally, the extraction will capture the study results, including qualitative and quantitative findings, along with the measurement instruments used. Two reviewers will independently select articles for review and extract data. Conflicts will be discussed, and a third reviewer will be consulted if a consensus cannot be reached. Descriptive statistics and thematic analysis (via NVivo) will be used to synthesize the findings.

**Results:**

This systematic literature review seeks to explore and synthesize existing research on how CMH technologies affect clinicians’ work environments and is expected to be completed by December 2025.

**Conclusions:**

This review will offer a comprehensive overview of how CMH technologies affect the professional work environment of clinicians.

**Trial Registration:**

PROSPERO CRD420251018685; https://www.crd.york.ac.uk/PROSPERO/view/CRD420251018685

**International Registered Report Identifier (IRRID):**

PRR1-10.2196/76668

## Introduction

### Overview

The global rise in mental health conditions has placed significant pressure on health care systems, with an increasing number of individuals seeking support [1]. Although not all individuals with the highest needs seek help due to factors such as stigma, cultural background, or socioeconomic status, those who do contribute to the growing demand for mental health care services [2,3]. This growing demand contributes to a heavier workload and greater responsibility for mental health professionals [4]. Research across various countries has highlighted that professionals working in mental health care often face some of the most challenging working conditions [5-7]. These include high workloads, elevated stress levels, administrative burdens, staffing shortages, limited resources, exposure to violence, interpersonal conflicts, and role ambiguity. It is also important to acknowledge that many mental health care professionals themselves experience burnout and mental health conditions. However, concerns about stigma and professional repercussions often make them reluctant to seek support [8]. Connected mental health (CMH) applications have been proposed as potential solutions to increase access to mental health care services and alleviate some of the pressures faced by mental health professionals [9,10].

CMH refers to the use of information and communication technologies in mental health care to overcome barriers such as stigma, education, and cost [11]. It encompasses various technology-based solutions, including online platforms, mobile apps, and wearable devices, which can aid in diagnosing, managing, and treating common mental health issues [12]. CMH has shown promise in assisting health care workers during the COVID-19 pandemic [11] and in supporting the transition from primary to secondary school [13]. Research indicates an increasing interest in CMH, with journals being the main publication channels and exploratory research dominating the field [11]. Although attitudes toward CMH are generally positive, concerns exist regarding technical issues, the digital divide, and institutional barriers [14].

The work environment is a multidimensional concept encompassing physical, social, and organizational factors that influence employee satisfaction, performance, and well-being [15,16]. It includes job demands, health management, work design, leadership, work-life balance, and recognition [16,17]. A positive work environment can improve organizational outcomes, reduce turnover, and increase engagement [18]. The workplace environment significantly impacts employee morale, productivity, and quality of life [19]. Organizations can enhance employee loyalty and well-being by improving working conditions and the overall environment [20]. Managers must create an atmosphere that attracts, retains, and motivates employees in today’s diverse and changing work landscape [18]. Digitalization has introduced both opportunities and challenges for the work environment, potentially enhancing efficiency and flexibility and increasing cognitive load, work intensification, and stress levels [21,22]. Although the specific impact of CMH technologies on work environments remains underexplored, it is important to recognize that mental health care already operates under highly stressful and pressured conditions [23]. Therefore, it is imperative to ensure that CMH implementations are designed to support and alleviate work-related stress rather than inadvertently contributing to additional strain on health care workers.

Several systematic reviews have examined the impact of mental health systems and workplace environments on the well-being of clinicians [24-26]. One major theme emerging from these reviews is the effectiveness of organizational-level interventions in reducing burnout and improving mental health among health care workers. A recent review emphasized that changes in job structure, work tasks, and physical environments are among the most effective strategies for supporting clinicians’ mental health [24]. In contrast, reviews of workplace mental health screening programs have shown mixed results. For example, a systematic review and meta-analysis found that screening followed only by advice or referral did not significantly improve outcomes, whereas screening combined with access to treatment produced small improvements [25]. Another review focusing on nurses in critical care settings found that the work environment was a significant contributor to high levels of depression and anxiety, pointing to the need for structural changes [26].

Although existing reviews provide valuable insights into the broader relationship between mental health systems and clinician well-being, they often focus on traditional organizational interventions, general workplace screening programs, or environmental stressors. However, there is a noticeable gap in synthesized knowledge specifically addressing how CMH technologies impact the work environment of mental health clinicians. The shift toward digital care has accelerated in recent years, particularly during and after the COVID-19 pandemic; however, the consequences for clinicians’ job demands, autonomy, workload, emotional labor, and professional identity remain fragmented across disciplines and underexplored in reviews. Moreover, although patient outcomes and accessibility are common focal points in CMH research, the clinician perspective, particularly in terms of occupational stress, job satisfaction, technology-induced strain, and ethical concerns, has not been systematically synthesized. This gap is especially critical given that mental health clinicians often manage emotionally intensive caseloads, and the integration of digital tools can both alleviate and exacerbate existing stressors.

In addition to traditional organizational interventions, a growing body of research has explored the use of digital technologies to address employee mental health in workplace settings. Web-based stress management programs and computer-assisted interventions have demonstrated benefits in reducing stress and enhancing coping skills among employees [27]. Workplace-delivered digital programs, including mobile apps for mindfulness, resilience, and psychological support, have been associated with improvements in well-being and reductions in anxiety and depression symptoms [28,29]. These studies suggest that technology-enabled interventions can be feasible and effective, but their adoption and sustained use often depend on usability, organizational readiness, and perceptions of confidentiality [29]. Although these advances illustrate the potential of digital tools to improve workplace mental health, evidence remains fragmented, and no systematic review has specifically examined how CMH technologies influence the work environment of mental health clinicians.

Therefore, this review aims to fill this critical gap by synthesizing the available evidence on the impact of CMH technologies on the work environment of mental health clinicians. It will offer insights into both the benefits and challenges of digital integration from a clinician-centered perspective, guiding technology design, policy, and workforce support in mental health services.

### Study Objectives

The objective of the proposed systematic review is to synthesize and critically analyze the reported outcomes and impacts of CMH technologies on mental health clinicians’ work environments across diverse global contexts.

### Research Questions

In the medical domain, the population, intervention, comparison, and outcome (PICO) framework is commonly used and recommended to develop research questions (RQs), especially for systematic literature reviews (Textbox 1) [30,31]. This ensures that only the studies most likely to be relevant will be retrieved and analyzed [32]. Although the PICO framework primarily aims to structure search strategies, past studies have occasionally excluded certain PICO elements based on research requirements such as the comparison component [33,34]. For this study, we also omit this component, as it is not suitable for the study. This is because we do not aim to rank the studies found or to compare them with some other existing approach.

**Population, intervention, comparison, and outcome structure.**

- Population: studies that include mental health care professionals such as psychiatrists and psychologists
- Intervention: connected mental health applications
- Comparison: not applicable
- Outcome: the work environment of mental health care professionals such as psychiatrists and psychologists

The population to be included will be mental health care professionals such as psychiatrists and psychologists. The intervention will be CMH applications. The outcomes of interest will be work-related stress or other work environment factors.

The following RQs guided this systematic literature review:

1. What are the positive and negative impacts of using CMH applications on mental health clinicians’ work environments?
2. What technical challenges are associated with the impact of CMH applications on mental health clinicians’ work environments?
3. Which design features of CMH applications enable their effectiveness in workplace settings for mental health clinicians?

In addition, we will report on the characteristics of the identified publications, including temporal distribution, publication avenues, country of origin, types of studies conducted, and types of CMH applications studied.

## Methods

### Study Design

This systematic review was submitted for registration with PROSPERO on April 23, 2025 (CRD420251018685) to avoid bias in conducting and reporting findings. According to the progress of the study, amendments will be made if necessary. The PRISMA-P (Preferred Reporting Items for Systematic Reviews and Meta-Analyses Protocols) checklist [35] is provided as Multimedia Appendix 1.

We selected a systematic literature review approach because our goal is to summarize the research related to the influence of CMH on the work environment and the barriers to adopting CMH, as well as to identify the types of mental health applications currently in use. Therefore, we followed widely recognized guidelines [30,31] to plan and conduct a systematic literature review. Database searches and screening were conducted between April and July 2025. Data extraction and analysis of the included studies are being performed between August and November 2025. The review manuscript is scheduled for submission in December 2025.

### Search Strategy

This review will analyze studies that discuss mental health care professionals’ use of CMH applications and their impact on the work environment. The search strategy for this systematic literature review was developed in collaboration with Görel Sundström, a librarian at Uppsala University, and the research team (SP, SO, MH, CB, ÅC, and MRS). The PICO statement was used to construct the search strategy, as shown in Table 1.

**Table 1:** Search strategy.

Search	Keyword group	Search string
1	Connected mental health	TITLE-ABS-KEY (“e mental health” OR “m mental health” OR ((“psychological health” OR “mental health” OR psychotherapy OR “behavi* therapy”) W/2 (connected OR digital OR mobile OR online OR smart OR tele OR video OR web)))
2	Work environment	TITLE-ABS-KEY (((job OR work*) W/2 (abuse* OR bullying OR condition* OR control* OR demand OR engagement OR environment* OR satisfaction OR stress* OR setting OR situation* OR strain OR violence OR well-being OR wellbeing)) OR “burn* out” OR burnout* OR “effort-reward imbalance” OR “employee* health” OR ergonomic* OR ERI OR “occupational health” OR “occupational safety” OR “occupational stress” OR “work life balance” OR “workplace health”)
3	Mental health professionals	TITLE-ABS-KEY (clinician* OR counsellor or counselor OR doctor* OR “health personnel” OR “health professional*” OR “healthcare professional*” OR “healthcare provider*” OR “healthcare worker*” OR “health care professional*” OR “health care provider*” OR “health care worker*” OR “general practitioner” OR “medical staff” OR nurse* OR “nursing staff” OR physician* OR psychiatrist* OR psychoanalyst* OR psychologist* OR psychotherapist* OR therapist*)
4	Combined search	1 AND 2 AND 3

The refinement of keywords involved multiple strategies: the librarian performed testing, and the team examined the cited articles to identify their keyword choices. The goal was to ensure that the search captured studies that addressed the same RQs using different terminologies.

The search will be conducted across a range of electronic databases: PubMed, IEEE Xplore, Scopus, and ACM Digital Library. The selected databases are chosen for their relevance to the research topic and their broad use in academic and research settings. This systematic review will not involve the collection of sensitive personal data. The search for publications is completed.

### Study Selection Criteria

The PICO statement was used to define the eligibility criteria for study inclusion and exclusion as shown in Table 2.

**Table 2:** Inclusion and exclusion criteria based on population, intervention, comparison, and outcome (PICO) framework.

PICO	Inclusion criteria	Exclusion criteria
Population	Mental health care professionals, physicians, and nurses, with no exclusion based on age or gender	Study participants who are not mental health care professionals, physicians, or nurses
Intervention	Mental health applications (eg, self-guided CBT^a^ apps, mood tracking, or AI^b^-driven chat support) and video consultations, which enable remote interaction between patients and mental health professionals.	General health care applications
Comparison	Not applicable	Not applicable
Outcome	Influence on the work environment	Not applicable
Study methods	Qualitative methods, quantitative methods, mixed methods, solution proposal, evaluation research, and experience articles	Reviews (systematic, scoping, meta-analysis, etc)
Publication types	Formally published peer-reviewed journal articles and conference articles	Gray literature, opinion pieces, protocols, and reviews

^a^CBT: cognitive behavioral therapy.

^b^AI: artificial intelligence.

### Types of Studies

#### Qualitative Studies

Qualitative studies include interviews, focus group discussions, usability studies, participatory research, participatory design, case studies, grounded theory research, thematic and content analysis of textual data, phenomenological studies, narrative research, and ethnographic observations.

#### Quantitative Studies

Quantitative studies include randomized controlled trials, cohort studies, longitudinal studies, experimental studies, case-control studies, cross-sectional studies, and observational studies.

#### Mixed Methods Studies

Mixed methods studies include those integrating qualitative and quantitative data collection and analysis methods within a single research design, encompassing, but not restricted to, convergent design, sequential explanatory design, and sequential exploratory design.

### Language

Because of limited resources, this study will only consider articles published in English. The research team recognizes that this decision restricts the inclusion of studies conducted in various regions and published in other languages.

### Study Screening

An independent librarian will first run a search using predefined keywords aligned with the inclusion and exclusion criteria to find potentially relevant studies. The search results will then be deduplicated by identifying and removing duplicates in EndNote (version 21; Clarivate), following the guidelines by Bramer et al [36]. The remaining studies will then be imported to Rayyan [37]. Data will first be screened on the title and abstract, and finally, on full-text screening. At least 2 reviewers will independently review the titles and abstracts during the first step. They will be blinded to each other’s review decision [38]. Thereafter, the potential articles will be screened based on their full text. In case of disagreements between the reviewers, another reviewer will be consulted to reach a consensus.

### Data Extraction

Data will be extracted using a standardized form developed by the research team. This form is structured to collect key study details, including (1) identification—study ID, authors, year, country, publication type, and study type (qualitative, quantitative, or mixed methods); (2) study characteristics—clinician demographics, age, sample size, and CMH application (digital platform or software used and delivery medium); and (3) results—findings in both qualitative and quantitative formats and the tools used for measurement.

Additional details may be incorporated if the research team deems them relevant to the analysis. One reviewer will carry out the data extraction independently, and a second reviewer will review the extracted data to ensure accuracy and completeness. All extracted information will be compiled into a predesigned table in Microsoft Excel. In addition, qualitative data related to the results and findings will be collected. These qualitative data will subsequently be analyzed to identify important themes using NVivo (version 14; Lumivero).

### Quality Assessment and Appraisal in the Screening Process

The quality of the included studies will be assessed using the Mixed Methods Appraisal Tool (MMAT) [39], which is specifically designed for systematic reviews that include qualitative, quantitative, and mixed methods studies. As shown in previous systematic reviews, the MMAT is reliable and efficient [40]. The assessment will be based on method-specific criteria, including the appropriateness and rigor of the methodology, control of confounding factors, minimization of selection bias, and consideration of study limitations. Two researchers will independently carry out the MMAT assessments and will reach a consensus on their evaluations. In cases of disagreement, a third researcher will be consulted.

### Data Analysis and Synthesis

**Figure 1:**
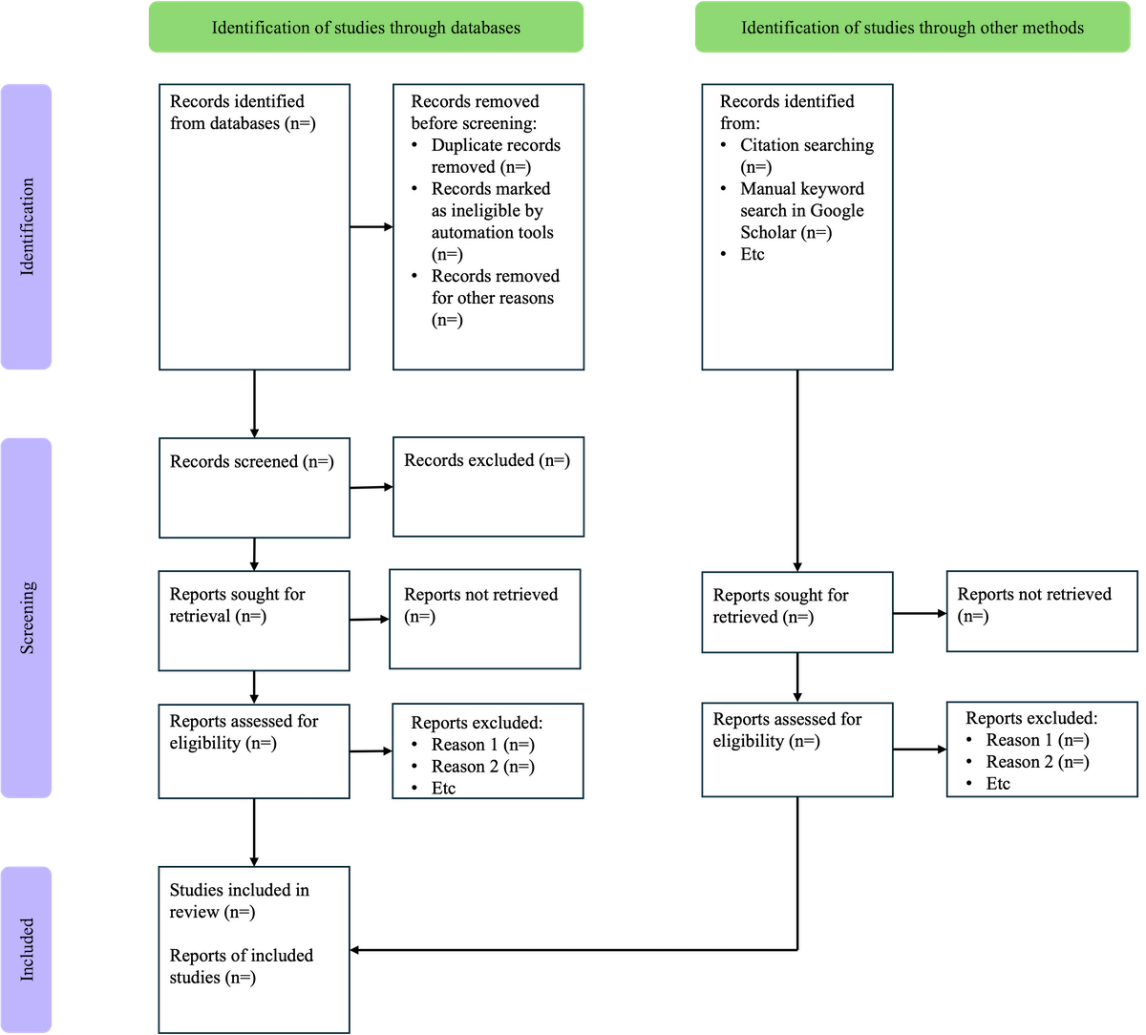
PRISMA (Preferred Reporting Items for Systematic Reviews and Meta-Analyses) flow diagram.

The study selection procedure will be illustrated using the PRISMA 2020 flow diagram, as demonstrated in Figure 1 [41]. Data synthesis will combine quantitative descriptive summaries with qualitative thematic analysis to ensure alignment with the RQs. These will be visualized through tables and charts in Microsoft Excel. Descriptive statistics will be used to address questions related to publication trends, study designs, contexts, and the types of CMH technologies investigated. This will involve frequency counts and distributions presented in tables and figures.

To address RQ1, we will conduct thematic analysis following the 6-phase framework proposed by Braun and Clarke [42]. Extracted data relating to work environment outcomes (eg, job demands, workload, autonomy, stress, and well-being) will be inductively coded. Codes will be collated into themes that represent how CMH technologies influence specific aspects of clinicians’ work environments. Themes will be reviewed and refined iteratively by multiple reviewers to ensure consistency, with NVivo software (version 14; Lumivero) used to support coding and transparency.

For RQ2, we will extract data relating to technical barriers (eg, interoperability, usability, system reliability, and data security). These will be coded inductively and synthesized thematically, highlighting how technical challenges shape or constrain work environment outcomes.

To answer RQ3, we will identify and code reported design features (eg, interface design, workflow integration, decision support, communication tools) and analyze them thematically. This synthesis will enable us to link specific design features to the reported positive or negative impacts in clinical work environments.

In addition to thematic analysis, descriptive statistics will summarize bibliometric and study characteristics (eg, year of publication, country, study design, type of CMH technology). These will contextualize the thematic findings and help identify patterns and gaps across the literature. Together, the descriptive and thematic analyses will provide a systematic answer to all 3 RQs.

One reviewer will conduct the coding, and a second reviewer will verify the sample for consistency. The findings will be synthesized narratively and supported by visual representations. The PRISMA 2020 flow diagram will illustrate the study selection process.

### Dissemination Strategy

The results of this study will be disseminated as a scientific publication in a peer-reviewed journal and presented at conferences. Results will also be shared on the blogs of various research groups that the authors are a part of.

### Ethical Considerations

According to the Ethical Review Act (2003:460) issued by the Swedish Ethical Review Authority, this research does not require ethics approval.

## Results

The authors have conducted 2 pilot searches to test and refine keywords and verify the initial quality of results with the help of the librarian. We have identified 386 studies for screening. Database searches and screening were completed by July 2025. The review team is currently engaged in data extraction and thematic analysis of the included studies, a process scheduled to be concluded by November 2025. The completed systematic review will be submitted for publication in December 2025.

## Discussion

### Anticipated Findings

This systematic review investigates how CMH applications influence the work environment of mental health clinicians. Although the results are pending, this review is expected to highlight the key trends, tools, outcomes, and challenges in this emerging field. We anticipate identifying a steady rise in publications, signaling a growing interest in CMH among researchers. Commonly studied technologies will likely include mobile apps and teletherapy platforms. Expected outcomes include positive effects such as reduced stress, improved mental well-being, and enhanced support for clinicians. However, the review also aims to uncover challenges such as poor usability, concerns over data privacy, digital fatigue, and limited institutional support, factors that may hinder adoption or long-term use. By focusing on clinicians, this review shifts attention from patient-centered outcomes to the well-being and work conditions of those delivering care. The findings will provide practical insights for developers, health care organizations, and policymakers seeking to implement CMH tools that genuinely support clinicians and, in turn, their patients, rather than compounding their burdens.

### Comparison to Previous Work

Previous reviews on digital workplace mental health programs have shown benefits for employee well-being, although adoption is shaped by usability and organizational readiness [27-29]. In contrast, our review focuses on clinicians as workers, examining how CMH tools used in patient care affect their own job demands and resources. Research on telemental health highlights implementation barriers, confidentiality, and increased electronic health record workload [43,44], while technostress studies describe how system complexity and poor support reduce well-being [22,45]. Our synthesis will connect these strands to clinicians’ work environments using the job demands–resources framework [46].

### Expected Contributions

This review is expected to make several contributions. First, it will provide a clinician-centered synthesis of how CMH applications influence work environment factors such as workload, autonomy, collaboration, and well-being—areas that are often overshadowed by patient-focused outcomes in the literature. Second, it will identify the design features and technical challenges most consistently associated with positive or negative impacts, offering insights into how these technologies can be optimized for clinical settings. Finally, by linking these findings to the job demands–resources framework, this review will generate practical guidance for both technology design and organizational implementation, thereby informing future development, policy, and research.

### Strengths, Limitations, and Future Directions

A significant strength of this review is that the protocol has been prospectively registered, which enhances transparency and reduces the risk of selective reporting. The review also follows PRISMA guidelines, ensuring methodological rigor in searching, screening, and reporting. Another strength is the use of 3 reviewers in screening and data extraction, which minimizes bias and increases reliability in study selection and coding. In addition, the combination of descriptive synthesis with thematic analysis provides both breadth—through mapping publication trends and study characteristics—and depth—through an interpretive account of how CMH technologies shape clinicians’ work environments. At the same time, several limitations of this study must be acknowledged. The included studies are likely to be heterogeneous in design, outcomes, and contexts, which may complicate the synthesis and limit comparability. Some studies may not include formal comparators, making it difficult to directly attribute changes to CMH technologies. Restricting the review to English-language studies also introduces the possibility of language bias, potentially excluding relevant work from non-English or gray literature sources. To mitigate these limitations, we will use transparent coding procedures, map outcomes consistently to the job demands–resources framework to enhance comparability and explicitly acknowledge contextual and methodological gaps in the evidence base.

### Conclusions

This protocol outlines a systematic literature review that will evaluate and synthesize evidence on the impacts of CMH technologies on the work environments of mental health clinicians. By systematically analyzing both positive and negative outcomes, technical challenges, and enabling design features, this review will provide an understanding of how CMH applications shape clinicians’ job demands, resources, and well-being. The use of a PRISMA-guided methodology, combined with descriptive and thematic synthesis, and alignment with the job demands–resources framework, supports the development of findings that will be robust and practically relevant. The anticipated contribution of this review is twofold: advancing the academic literature by clarifying the state of evidence in this area and informing technology developers, health care organizations, and policymakers seeking to design and implement CMH solutions that are sustainable and supportive of clinicians’ work environments. Ultimately, the results are expected to generate insights that can guide future research, design, and policy, while also identifying critical gaps, particularly in underrepresented regions, that warrant further investigation.

## Appendix

Multimedia Appendix 1 PRISMA-P checklist.

## Abbreviations

CMH: connected mental health
MMAT: Mixed Methods Appraisal Tool
PICO: population, intervention, comparison, and outcome
PRISMA-P: Preferred Reporting Items for Systematic Reviews and Meta-Analyses Protocols
RQ: research question

## Data Availability

Data sharing is not applicable to this study as no datasets were generated or analyzed during this study.

## References

[1] Vigo D, Thornicroft G, Atun R (2016). Estimating the true global burden of mental illness. Lancet Psychiatry.

[2] Andrade LH, Alonso J, Mneimneh Z, Wells JE, Al-Hamzawi A, Borges G, Bromet E, Bruffaerts R, de Girolamo G, de Graaf R, Florescu S, Gureje O, Hinkov HR, Hu C, Huang Y, Hwang I, Jin R, Karam EG, Kovess-Masfety V, Levinson D, Matschinger H, O'Neill S, Posada-Villa J, Sagar R, Sampson NA, Sasu C, Stein DJ, Takeshima T, Viana MC, Xavier M, Kessler RC (2014). Barriers to mental health treatment: results from the WHO World Mental Health surveys. Psychol Med.

[3] Clement S, Schauman O, Graham T, Maggioni F, Evans-Lacko S, Bezborodovs N, Morgan C, Rüsch N, Brown JS, Thornicroft G (2015). What is the impact of mental health-related stigma on help-seeking? A systematic review of quantitative and qualitative studies. Psychol Med.

[4] Yang Y, Hayes JA (2020). Causes and consequences of burnout among mental health professionals: a practice-oriented review of recent empirical literature. Psychotherapy (Chic).

[5] Hylén U, Kjellin L, Pelto-Piri V, Warg LE (2018). Psychosocial work environment within psychiatric inpatient care in Sweden: violence, stress, and value incongruence among nursing staff. Int J Ment Health Nurs.

[6] Cosgrave C, Maple M, Hussain R (2018). Work challenges negatively affecting the job satisfaction of early career community mental health professionals working in rural Australia: findings from a qualitative study. J Ment Health Train Educ Pract.

[7] O'Connor K, Muller Neff D, Pitman S (2018). Burnout in mental health professionals: a systematic review and meta-analysis of prevalence and determinants. Eur Psychiatry.

[8] Knaak S, Mantler E, Szeto A (2017). Mental illness-related stigma in healthcare: barriers to access and care and evidence-based solutions. Healthc Manage Forum.

[9] Naslund JA, Aschbrenner KA, Araya R, Marsch LA, Unützer J, Patel V, Bartels SJ (2017). Digital technology for treating and preventing mental disorders in low-income and middle-income countries: a narrative review of the literature. Lancet Psychiatry.

[10] Hollis C, Morriss R, Martin J, Amani S, Cotton R, Denis M, Lewis S (2015). Technological innovations in mental healthcare: harnessing the digital revolution. Br J Psychiatry.

[11] Drissi N, Ouhbi S, Janati Idrissi MA, Fernandez-Luque L, Ghogho M (2020). Connected mental health: systematic mapping study. J Med Internet Res.

[12] Roberts LW, Chan S, Torous J (2018). New tests, new tools: mobile and connected technologies in advancing psychiatric diagnosis. NPJ Digit Med.

[13] Lester L, Waters S, Cross D (2013). The relationship between school connectedness and mental health during the transition to secondary school: a path analysis. Aust J Guid Couns.

[14] Drissi N, Ouhbi S, Serhani MA, Marques G, de la Torre Díez I (2023). Connected mental health solutions: global attitudes, preferences, and concerns. Telemed J E Health.

[15] Suleiman AM (2023). Exploring work environment management boundaries using work domain analysis. Saf Sci.

[16] Greig MA, Searcy C, Neumann WP (2021). Work environment in the context of corporate social responsibility reporting: developing common terms for consistent reporting in organizations. J Clean Prod.

[17] Maassen SM, van Oostveen C, Vermeulen H, Weggelaar AM (2021). Defining a positive work environment for hospital healthcare professionals: a Delphi study. PLoS One.

[18] Badrianto Y, Ekhsan M (2020). Effect of work environment and job satisfaction on employee performance in PT. Nesinak Industries. J Bus Manag Account.

[19] Edem MJ, Akpan EU, Pepple NM (2017). Impact of workplace environment on health workers. Occup Med Health Aff.

[20] Gorgenyi-Hegyes E, Nathan RJ, Fekete-Farkas M (2021). Workplace health promotion, employee wellbeing and loyalty during Covid-19 pandemic—large scale empirical evidence from Hungary. Economies.

[21] Messenger J, Llave OV, Gschwind L, Boehmer S, Vermeylen G, Wilkens M (2017). Working anytime, anywhere: the effects on the world of work. European Foundation for the Improvement of Living and Working Conditions.

[22] Tarafdar M, Pullins EB, Ragu‐Nathan TS (2014). Technostress: negative effect on performance and possible mitigations. Information Systems Journal.

[23] Ruotsalainen JH, Verbeek JH, Mariné A, Serra C (2014). Preventing occupational stress in healthcare workers. Cochrane Database Syst Rev.

[24] Aust B, Leduc C, Cresswell-Smith J, O'Brien C, Rugulies R, Leduc M, Dhalaigh DN, Dushaj A, Fanaj N, Guinart D, Maxwell M, Reich H, Ross V, Sadath A, Schnitzspahn K, Tóth MD, van Audenhove C, van Weeghel J, Wahlbeck K, Arensman E, Greiner BA, MENTUPP consortium members (2024). The effects of different types of organisational workplace mental health interventions on mental health and wellbeing in healthcare workers: a systematic review. Int Arch Occup Environ Health.

[25] Strudwick J, Gayed A, Deady M, Haffar S, Mobbs S, Malik A, Akhtar A, Braund T, Bryant RA, Harvey SB (2023). Workplace mental health screening: a systematic review and meta-analysis. Occup Environ Med.

[26] Wells SK (2024). The impact of nurses' work environment on mental health and suicide. Crit Care Nurse.

[27] Heber E, Ebert DD, Lehr D, Cuijpers P, Berking M, Nobis S, Riper H (2017). The benefit of web- and computer-based interventions for stress: a systematic review and meta-analysis. J Med Internet Res.

[28] Joyce S, Modini M, Christensen H, Mykletun A, Bryant R, Mitchell PB, Harvey SB (2016). Workplace interventions for common mental disorders: a systematic meta-review. Psychol Med.

[29] Carolan S, Harris PR, Cavanagh K (2017). Improving employee well-being and effectiveness: systematic review and meta-analysis of web-based psychological interventions delivered in the workplace. J Med Internet Res.

[30] Kitchenham B, Charters S (2007). Guidelines for performing systematic literature reviews in software engineering: version 2.3. EBSE Technical Report.

[31] Petticrew M, Roberts H (2008). Systematic Reviews in the Social Sciences: A Practical Guide.

[32] Greenes RA (2007). Clinical Decision Support: The Road Ahead.

[33] Oriol M, Marco J, Franch X (2014). Quality models for web services: a systematic mapping. Inf Softw Technol.

[34] James KL, Randall NP, Haddaway NR (2016). A methodology for systematic mapping in environmental sciences. Environ Evid.

[35] Shamseer L, Moher D, Clarke M, Ghersi D, Liberati A, Petticrew M, Shekelle P, Stewart LA, PRISMA-P Group (2015). Preferred reporting items for systematic review and meta-analysis protocols (PRISMA-P) 2015: elaboration and explanation. BMJ.

[36] Bramer WM, Giustini D, de Jonge GB, Holland L, Bekhuis T (2016). De-duplication of database search results for systematic reviews in EndNote. J Med Libr Assoc.

[37] Rayyan: faster systematic literature reviews. Rayyan.

[38] Harrison H, Griffin SJ, Kuhn I, Usher-Smith JA (2020). Software tools to support title and abstract screening for systematic reviews in healthcare: an evaluation. BMC Med Res Methodol.

[39] Hong QN, Pluye P, Fàbregues S, Bartlett G, Boardman F, Cargo M, Dagenais P, Gagnon MP, Griffiths F, Nicolau B, O’Cathain A, Rousseau MC, Vedel I (2018). Mixed Methods Appraisal Tool (MMAT) version 2018. McGill University.

[40] Pace R, Pluye P, Bartlett G, Macaulay AC, Salsberg J, Jagosh J, Seller R (2012). Testing the reliability and efficiency of the pilot Mixed Methods Appraisal Tool (MMAT) for systematic mixed studies review. Int J Nurs Stud.

[41] Page MJ, McKenzie JE, Bossuyt PM, Boutron I, Hoffmann TC, Mulrow CD, Shamseer L, Tetzlaff JM, Akl EA, Brennan SE, Chou R, Glanville J, Grimshaw JM, Hróbjartsson A, Lalu MM, Li T, Loder EW, Mayo-Wilson E, McDonald S, McGuinness LA, Stewart LA, Thomas J, Tricco AC, Welch VA, Whiting P, Moher D (2021). The PRISMA 2020 statement: an updated guideline for reporting systematic reviews. BMJ.

[42] Braun V, Clarke V (2008). Using thematic analysis in psychology. Qual Res Psychol.

[43] Lawrence K, Nov O, Mann D, Mandal S, Iturrate E, Wiesenfeld B (2022). The impact of telemedicine on physicians' after-hours electronic health record "work outside work" during the COVID-19 pandemic: retrospective cohort study. JMIR Med Inform.

[44] Shore JH, Yellowlees P, Caudill R, Johnston B, Turvey C, Mishkind M, Krupinski E, Myers K, Shore P, Kaftarian E, Hilty D (2018). Best practices in videoconferencing-based telemental health April 2018. Telemed J E Health.

[45] Tarafdar M, Cooper CL, Stich JF (2017). The technostress trifecta ‐ techno eustress, techno distress and design: theoretical directions and an agenda for research. Info Systems J.

[46] Bakker AB, Demerouti E (2017). Job demands-resources theory: taking stock and looking forward. J Occup Health Psychol.

